# A Methodological Refinement for Evaluating Adoption of Priority Practices for Water Quality Improvement in Australia’s Great Barrier Reef

**DOI:** 10.1007/s00267-026-02449-6

**Published:** 2026-04-21

**Authors:** Geoff Kuehne, Will Higham, Kevin McCosker, Adam Northey, Rob Hassett, Emily Brooks, Paul Humphreys

**Affiliations:** 1 Meaningful Social Research, Balhannah, SA Australia; 2 Adelaide University, Adelaide, SA Australia; 3 Farmacist, Cairns, QLD Australia; 4 Department of Primary Industries, Brisbane, QLD Australia

**Keywords:** ADOPT tool, Perspective-taking, Farmer decision-making, Adoption modelling, Agricultural extension, Great Barrier Reef

## Abstract

Efforts to improve Australia’s Great Barrier Reef (GBR) water quality are constrained less by a lack of identified priority practices than by their persistent low and uneven adoption by farmers. Existing prioritisation and target-setting approaches largely emphasize biophysical effectiveness, with a limited focus on how farmers perceive and respond to proposed practice changes. This study introduces a methodological refinement of ADOPT (Adoption and Diffusion Outcome Prediction Tool) that embeds explicit, facilitated perspective-taking into adoption prediction. Rather than treating the use of ADOPT as a purely technical exercise, the approach uses expert-guided workshops in which Technical Working Groups respond to ADOPT questions from the perspective of the target population of farmers. This process generates baseline and ceiling adoption predictions that are grounded in farmers’ decision-making realities. Using the priority practice of a dual-herbicide sprayer for sugarcane farming—a technology that applies different herbicides to different parts of each cane row, allowing some toxic residual herbicides to be replaced with less toxic alternatives—as a case example, we show that although perspective-taking is effortful and requires facilitation, it improves the credibility and realism of predicted adoption outcomes. The approach also clarifies which adoption influences are most constraining and where policy and programme interventions are most likely to be effective. This method provides policymakers with a transparent, cost-effective, and defensible basis for setting realistic practice-change targets, prioritising investments, and improving the effectiveness of GBR water quality programmes.

## Introduction

**Fig. 1:**
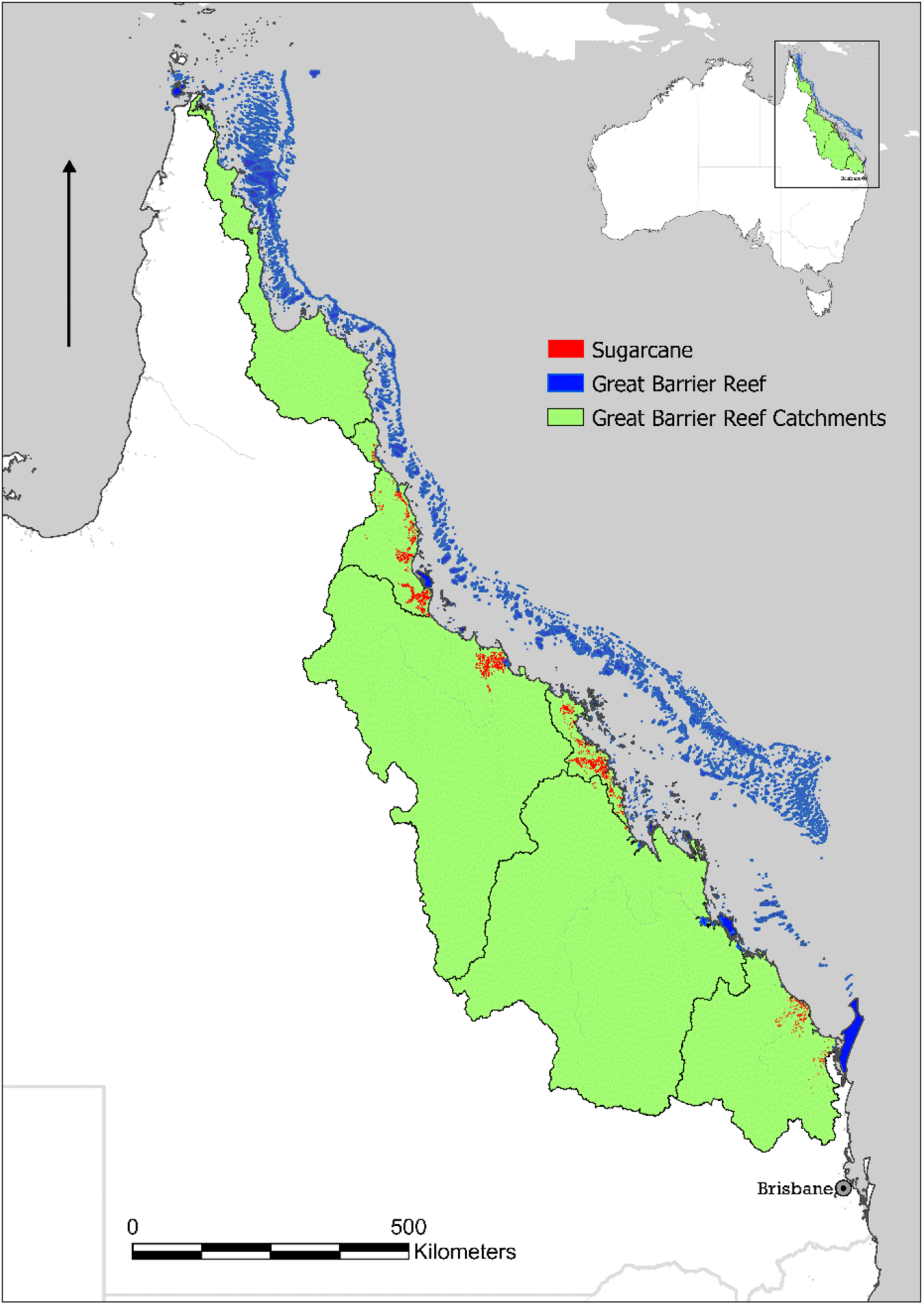
Australia’s Great Barrier Reef, catchment area, and sugarcane area. Base map source: Esri, CGIAR, USGS; data sources: Esri, TomTom, Garmin, FAO, NOAA, USGS, © OpenStreetMap contributors, and the GIS User Community

Improving water quality in the Great Barrier Reef (GBR) catchment in Queensland, Australia (Fig. 1), remains a significant national and global conservation and management challenge, requiring robust, transparent, and scientifically defensible approaches for prioritising land-use and catchment management practices. This article reports on the process used to develop priority landscape practices, one of the five actions identified by the Australian Government for improving Great Barrier Reef water quality (Commonwealth of Australia 2023). While agriculture is the dominant land use in the GBR catchment and the largest contributor to water pollution, the Reef is also affected by urban stormwater, industrial discharge and sewage treatment outfalls, coastal development, land clearing, marine debris, as well as dredging, ports and shipping, mining, and other land and water-based pressures (Commonwealth of Australia 2023; State of Queensland, 2018; Wang et al. 2020; Saint-Amand et al. 2022). The management practices of farmers in catchments adjacent to the GBR nevertheless play a crucial role in reef health, as runoff from agricultural activities contributes sediment, fertilizer and pesticide pollution that negatively affects reef ecosystems (Kroon et al. 2016; Haynes et al. 2007).

Priority practices are land management practices prioritized for their expected cost-effective contribution to reducing pollutant loads to the GBR, based on their water quality benefits and current levels of adoption (Thorburn et al. 2011).

Despite widespread awareness of practices promoted under the Reef 2050 Water Quality Improvement Plan (WQIP), adoption has often been limited or inconsistent (The State of Queensland, 2024). The adoption of many practices remains insufficient and highly variable across catchments and industries, with consequences for the long-term health of the GBR (Coggan et al. 2021). Where non-adoption or low adoption occurs, these outcomes can often be understood as rational decisions when considered from the farmer’s perspective, reflecting assessments of risk, cost, effort, and system fit rather than a lack of awareness or willingness to change (Vanclay 2004).

**Fig. 2:**
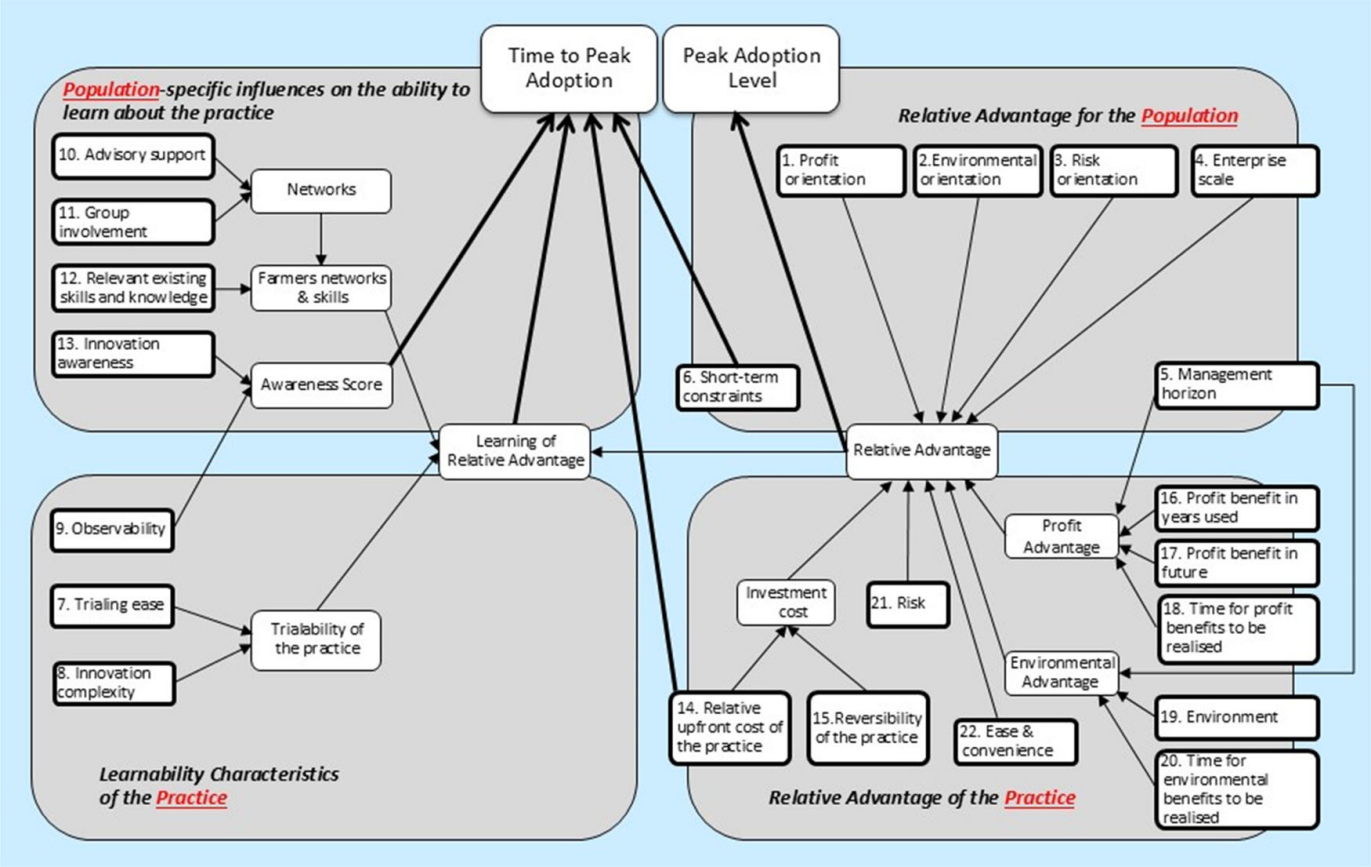
Conceptual framework of the ADOPT tool

Everett Rogers (2003, p.15) defined relative advantage as “the degree to which an innovation is perceived as being better than the idea it supersedes.” Kuehne et al. (2017) operationalized relative advantage within the ADOPT framework (Fig. 2) as the weighted aggregation of perceived economic, environmental, risk, and management effects of an innovation, moderated by the motivational orientations of the target population. Analysis using the ADOPT^1^ tool (Kuehne et al. 2017), which predicts adoption outcomes based on responses to 22 structured adoption influence questions, indicates that many proposed priority practices exhibit low or negligible relative advantage compared with the practices they are intended to replace (Higham et al. 2025). Under such conditions, low adoption rates are to be expected (Rogers 2003). Practices aimed at reducing sediment, fertilizer, or pesticide impacts to the GBR often provide limited direct on-farm benefits in terms of short-term profitability, risk reduction, or ease of management. While these practices may deliver substantial public environmental benefits, their relatively weak private advantages in the short-term help explain the persistent difficulty of achieving widespread uptake.

Despite this, many priority practices continue to be promoted with optimistic assumptions regarding their likely rate and extent of adoption, even where past efforts have achieved limited uptake. Evidence suggests that more effective strategies are likely to emerge when interventions are tailored to specific practices, emphasize benefits that matter to farmers, and align with landholders’ broader goals and decision-making contexts (Rolfe and Gregg 2015). Achieving this requires recognizing that when the stakes are high, as they are for GBR water quality, greater rigour in analysis, consultation, and reflection is warranted. The scale of potential consequences associated with unsuccessful practice change should determine the level of analytical effort applied to education, consultation, prioritisation and target setting.

Developing priority practices for water quality management therefore necessarily involves perspective-taking, as it requires understanding how farmers perceive the risks, constraints, and benefits associated with scientifically recommended practices. However, existing prioritisation frameworks have predominantly emphasized biophysical effectiveness and cost-efficiency, with limited systematic integration of farmer perspectives into the ranking and target-setting process. As a result, priority practices may be identified as technically effective and subsequently promoted without sufficient consideration of whether they are likely to be adopted at the scale or within the timeframes required to achieve programme objectives.

These issues are especially salient when using adoption prediction tools such as ADOPT. ADOPT is one of the few ready-to-use tools developed specifically for agricultural research and development planning and extension forecasting. It evaluates 22 adoption influences across four domains: innovation characteristics, learning characteristics, social characteristics, and landholder characteristics. These are used to generate ex ante predictions of Peak Adoption Levels and Time to Peak Adoption (Kuehne et al. 2017). Despite the breadth of established adoption models in the literature, ADOPT is distinctive in being explicitly designed to generate predictive numerical adoption estimates in agriculture (Montes de Oca Munguia et al. 2021). Because ADOPT relies on qualitative judgements provided by informed users, the extent to which those judgements accurately reflect how the target population is likely to perceive and respond to a proposed innovation directly influences the reliability of its predictions.

Although ADOPT could draw on data from statistically representative farmer surveys or interviews, such approaches are typically better suited to in-depth analysis of a small number of practices within a defined population. In this study, ADOPT was applied to prioritize adoption prospects across 86 candidate practices, making practice-specific farmer surveys impractical within programme constraints. Moreover, ADOPT requires structured judgements about how non-adopting farmers would perceive and experience counterfactual adoption conditions—assessments that can be difficult to elicit directly when practices are novel or not yet familiar within the farming community. We therefore used a facilitated perspective-taking process to enhance the representativeness of the inputs and reduce the risk of expert-centric bias.

However, perspective-taking is not a simple or automatic process. It involves actively considering another person’s point of view and how they perceive their circumstances, constraints, and options (Mark Davis 1983; Galinsky and Ku 2004). This process is effortful and subject to cognitive limitations, including anchoring on one’s own perspective and the need for deliberate adjustment to approximate another’s viewpoint (Epley and Gilovich 2001; Epley et al. 2004). These challenges are particularly relevant for scientists, policymakers, and technical experts whose professional frames of reference differ substantially from those of the farmers whose decisions they seek to anticipate (Mark Davis et al. 1996). Without structured support, attempts at perspective-taking may remain partial or be influenced by assumptions about what farmers should do rather than how they are most likely to respond.

To improve the credibility and usefulness of adoption predictions informing GBR water quality programmes, we developed a method that situates the application of ADOPT within a structured perspective-taking process. Rather than treating ADOPT as a purely technical exercise, this approach explicitly incorporates consideration of farmers’ local realities and decision-making contexts. Assessing adoption outcomes across a large and diverse portfolio of practices requires informed judgment that integrates technical knowledge with an understanding of farmer behaviour. Technical Working Groups (TWGs), comprising experts from research, policy, extension, and practical farming, provide a structured mechanism for bringing these perspectives together. Many of TWG participants are professionally embedded within the industries concerned and therefore hold a shared interest in the long-term viability and performance of those industries. While this may shape their perspectives, it also reflects the institutional and economic realities within which priority practices must operate. Adoption targets that are disconnected from industry conditions, constraints, and incentives are unlikely to be credible or implementable. Rather than attempting to suppress embedded industry perspectives, the workshop design can seek to make underlying assumptions explicit and subject them to structured consideration. During workshop, TWG members can be encouraged to reflect on how farmers’ perceptions and constraints would shape likely adoption decisions when formulating responses to the ADOPT questions. This guided perspective-taking process requires participants to respond from the standpoint of non-adopting farmers rather than from personal or organisational positions. By embedding these discussions within ADOPT’s structured question set, the process reduces the scope for unexamined advocacy and focused deliberation on clearly defined adoption influences. This article describes the development and application of this methodological refinement to generate priority practice adoption targets that are more closely aligned with real-world farmer decision-making.

The objectives of this study were to:

1. Apply the ADOPT tool using an explicit perspective-taking approach, whereby responses to the ADOPT questions reflect the perceived perspectives of the target population of farmers.
2. Develop a baseline prediction of priority practice adoption outcomes, representing expected adoption in the absence of additional intervention.
3. Develop a ceiling prediction of priority practice adoption outcomes, representing expected adoption under a well-resourced intervention scenario.

The Dual Band Sprayer in sugarcane farming is presented as a case example to illustrate the application of this refined ADOPT method.

## Methodology

The following section outlines the methodological approach used in this study, including the application of the ADOPT tool, the workshop process, and the composition and role of the Technical Working Groups.

### The ADOPT Tool

ADOPT provides a structured analysis of how a group of farmers make decisions about adopting an innovation by examining the key factors that influence adoption decisions (Kuehne et al. 2017). It does this by guiding users through a set of 22 structured questions linked to adoption influences (Table 1). The tool is applied as a methodological and decision-analytic framework, rather than as a behavioural experiment, and does not involve direct observation or manipulation of farmer decision-making. Consequently, ADOPT is not intended to predict individual farmer adoption choices or to replace empirical adoption studies, instead it provides ex ante estimates of adoption outcomes that can be used to inform policy and programme design. In doing so, ADOPT helps users develop an evidence-based assessment of likely Peak Adoption Levels and Time to Peak Adoption. In addition, it generates a sensitivity analysis identifying which adoption influences have the greatest effect on predicted outcomes by showing the impact of a one-step change in a single influence while holding all others constant. These outputs allow users to develop insights into how the characteristics of the innovation, the target population, and the broader decision context interact to shape adoption, and to identify constraints and leverage points for improving adoption outcomes.

**Table 1:** The ADOPT questions

1. Profit orientation	What proportion of the target population has maximising profit as a strong motivation?
2. Environmental orientation	What proportion of the target population has protecting the natural environment as a strong motivation?
3. Risk orientation	What proportion of the target population has risk minimisation as a strong motivation?
4. Enterprise scale	On what proportion of the target farms is there a major enterprise that could benefit from the innovation?
5. Management horizon	What proportion of the target population has a long-term (greater than 10 years) management horizon for their farm?
6. Short term constraints	What proportion of the target population is under conditions of severe short-term financial constraints?
7. Trialable	How easily can the innovation (or significant components of it) be trialled on a limited basis before a decision is made to adopt it on a larger scale?
8. Innovation complexity	Does the complexity of the innovation allow the effects of its use to be easily evaluated when it is used?
9. Observability	To what extent would the innovation be observable to farmers who are yet to adopt it when it is used in their district?
10. Advisory support	What proportion of the target population uses paid advisors capable of providing advice relevant to the project?
11. Group involvement	What proportion of the target population participates in farmer- based groups that discuss farming?
12. Relevant existing skills & knowledge	What proportion of the target population will need to develop substantial new skills and knowledge to use the innovation?
13. Innovation awareness	What proportion of the target population would be aware of the use or trialling of the innovation in their district?
14. Relative upfront cost of the project	What is the size of the up. front cost of the investment relative to the potential annual benefit from using the innovation?
15. Reversibility of the innovation	To what extent is the adoption of the innovation able to be reversed?
16. Profit benefit in years that it is used	To what extent is the use of the innovation likely to affect the profitability of the farm business in the years that it is used?
17. Future profit benefit	To what extent is the use of the innovation likely to have additional effects on the future profitability of the farm business?
18. Time until any future profit benefits are likely to be realised	How long after the innovation is first adopted would it take for effects on future profitability to be realised?
19. Environmental costs & benefits	To what extent would the use of the innovation have net environmental benefits or costs?
20. Time to environmental benefit	How long after the innovation is first adopted would it take for the expected environmental benefits or costs to be realised?
21. Risk exposure	To what extent would the use of the innovation affect the net exposure of the farm business to risk?
22. Ease and convenience	To what extent would the use of the innovation affect the ease and convenience of the management of the farm in the years that it is used?

Like all models, ADOPT’s predictions are subject to inherent limitations. Uncertainty arises because the tool relies on qualitative user judgements that are recorded on Likert-type ordinal scales, where responses may fall between scale points and the intervals between points are unlikely to be uniform. In effect, translating qualitative assessments into quantitative inputs introduces a measure of uncertainty. This uncertainty is characteristic of models of this type and highlights the importance of using the most accurate and representative inputs possible to maximize the reliability of ADOPT’s predictions.

### The Workshops

The ADOPT workshop was designed to contribute to the formulation of credible and defensible adoption targets for priority farm management practices to inform the forthcoming Reef 2050 Water Quality Improvement Plan 2025–30. Given that the process of establishing priority practice targets entails significant social risks such as legitimacy, equity, and stakeholder trust, it is essential that it be undertaken with methodological rigour and analytical robustness. Without this, there is a risk that farmers and industries will be provided with unrealistic or unachievable targets and then assessed on how they have met them. Such targets, if perceived as out of touch with the practical realities of farming, can generate frustration and disillusionment, reinforce perceptions of unfairness, and foster distrust of government intentions.

At the same time, substantial practice change at landscape scales is likely to be necessary to achieve meaningful improvements in water quality and Great Barrier Reef health within required timeframes. The challenge will not be avoiding ambitious targets, but ensuring they are grounded in realistic assessments of adoption potential and accompanied by appropriately resourced intervention strategies. Optimistic targets may be justified where credible pathways to change exist, including extension, incentives, and industry engagement. In this context, the role of tools such as ADOPT is to clarify the likely baseline adoption outcomes and to identify the scale and nature of intervention that needs to be required if higher adoption levels are to be achieved.

Importantly, the ADOPT workshop process focused specifically on estimating voluntary adoption of priority practices beyond existing regulatory minimum standards. The intention was to assess likely uptake under prevailing policy settings. Regulation forms part of the broader decision environment within which farmers operate, but the ADOPT modelling exercise was concerned with voluntary practice change, as this is where uncertainty about adoption outcomes is greatest and where adoption targets are most sensitive to overestimation.

Fifteen workshops were conducted over eight weeks in thirteen locations across the farming areas of the GBR to examine the likely adoption outcomes of existing practices for improved water quality and the potential for increasing their uptake. The workshops examined a diverse set of priority practices that varied by both industry and catchment context across the five key GBR agricultural industries—bananas, grains, grazing, horticulture, and sugarcane. As part of the WQIP process, our intention was not merely to trial a perspective-taking approach to the use of the ADOPT tool, but to implement it in practice. The aim was to refine the approach so that priority practice adoption predictions more accurately reflect real-world decision-making conditions.

**Fig. 3:**
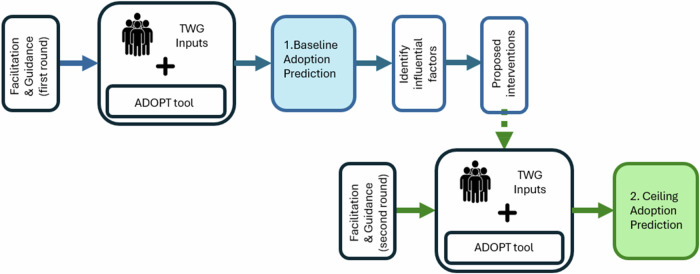
The structured process of generating baseline and ceiling predictions of adoption outcomes with the technical working group (TWG)

The workshops engaged TWG members in a structured process (Fig. 3) to identify and describe priority practice changes and the non-adopters to whom they were most relevant. The workshops followed a structured ADOPT process in which participants first defined and ranked priority practices for their industry and region. For example, within the sugarcane industry, one of the priority practices examined, and used here as a case example, was the dual band herbicide sprayer. Participants specified what the practice included and excluded, and documented the relevant history of the innovation, current adoption levels, and the characteristics of the target population of non-adopters for whom the practice was relevant and realistically adoptable. Participants then developed baseline adoption predictions using current knowledge, considered the key drivers influencing those outcomes, and identified which drivers could plausibly be changed. Using ADOPT’s sensitivity analysis, the most influential adoption factors were identified and discussed, and participants proposed interventions designed to modify these factors. These interventions were incorporated into the baseline scenarios to generate ceiling adoption predictions, representing potential adoption outcomes under well-resourced intervention conditions. Further details for the dual band herbicide sprayer case are available in the “Dual Herbicide Sprayer Ceiling Adoption Report” (PDF), which presents the full printed output of the ADOPT tool analysis (Supplementary Information).

The workshops were facilitated by the second author, with the first author guiding participants through the ADOPT process. Each workshop commenced with an introduction to the overall project and workshop objectives, followed by discussion and prioritisation of the previously identified priority practices. Workshop discussions were used to elicit agreed responses to the predefined 22 ADOPT questions, which were recorded directly in the tool during the sessions. These inputs were analyzed using ADOPT’s internal modelling and sensitivity analysis functions; no separate qualitative analysis was undertaken (Fig. 3).

While regulation is an important driver of practice change in the GBR context, the ADOPT workshop process in this study focused specifically on voluntary adoption of priority practices beyond existing regulatory requirements. The intention was not to model compliance behaviour, but to estimate likely uptake under current policy settings. Regulatory frameworks nonetheless form part of the broader decision environment and may influence several ADOPT inputs indirectly (e.g., perceived risk, relative advantage, and reversibility), particularly where practices move from voluntary to mandatory over time.

### The Technical Working Groups

Fifteen regional Technical Working Groups (TWGs) were established by the Queensland Department of Agriculture and Fisheries Management Practice Adoption (MPA) team for each priority agricultural industry—banana, grains, grazing, horticulture, and sugarcane—across the GBR regions of Cape York, Wet Tropics, Burdekin, Mackay Whitsunday, Fitzroy, and Burnett Mary. Membership was identified in consultation with industry and research partners, with experts and specialists selected to represent specific industries in their regions. In total, 92 TWG representatives participated in the workshop. Collectively, the TWGs refined and prioritized 86 discrete priority practices using the ADOPT model to assess their potential to accelerate water quality improvements in priority catchments.

Participants were not selected to be statistically representative of the broader stakeholder community. Instead, TWG membership was purposively selected to include individuals with complementary expertise across research, policy, extension, and farming systems, and with sustained professional engagement with the target populations. This combination of expertise allowed them to make informed judgements about farmers’ likely perceptions, constraints, and responses to priority practices, grounded in both the scientific and policy foundations of the practices and an understanding of growers’ day-to-day realities.

Prior to using the ADOPT tool in the workshops, participants were explicitly asked to frame their responses to the ADOPT questions from the perspective of the target population of farmers. This combination of expertise and structured perspective-taking enabled the TWGs to bridge the gap between theoretical discussions of priority practices and farmers’ needs for practical, implementable solutions.

## Results

Our application of the ADOPT tool was designed to support participants in providing responses to the ADOPT questions that most accurately reflected how the target population would respond. This approach was intended to improve the reliability of the ADOPT tool by making its predictions more closely aligned with how farmers are likely to respond to the factors influencing adoption of priority practices. TWG members were instructed to frame their responses to the ADOPT questions from the perspective of the target population, and participants were reminded throughout the workshop to maintain this perspective when answering the questions. To illustrate how the refined ADOPT process was applied in practice, a worked example from one workshop is presented below.

### Case Example: The Dual Herbicide Sprayer

The Dual Herbicide Sprayer serves as an illustrative case through which the refined ADOPT workshop process is demonstrated. The emphasis is methodological rather than technological; the purpose is to show how adoption predictions are generated and refined, not to provide a comprehensive technical evaluation of the innovation. The sprayer is a targeted spraying technology designed for sugarcane production that applies different herbicides to different parts of each cane row, enabling some toxic residual herbicides used across the entire row to be replaced with less toxic alternatives. This example is drawn from the sugarcane workshop held in Ingham on 10 May 2024, which involved six TWG participants representing the sugarcane industry in the Wet Tropics South region.

Sugarcane farming occupies approximately 400,000 hectares within the GBR catchment (Fig. 1). Although this represents only about 1.4% of the total catchment area, it is a disproportionately high-impact land use. Sugarcane-growing regions contribute an estimated 78% of anthropogenic dissolved inorganic nitrogen (DIN) loads and more than 95% of pesticide loads delivered to the Reef. These pollutants are strongly associated with algal blooms, crown-of-thorns starfish outbreaks, reduced coral diversity, and increased susceptibility of corals to bleaching and disease (State of Queensland 2018). In this context, adoption outcomes for priority practices within the sugarcane sector are critically important for achieving GBR water quality objectives.

Within this broader environmental and policy context, the Dual Herbicide Sprayer was selected as a suitable illustrative practice. It represents a relatively simple and clearly defined innovation within a high-profile GBR industry. Adoption does not require substantial restructuring of the broader farming system, yet existing uptake remains low, providing scope for improvement. These characteristics make it well suited to demonstrating the application of the refined ADOPT process.

### TWG-Informed ADOPT Assessment

To demonstrate how the refined ADOPT process was applied, the TWG first articulated the problem the innovation was intended to address and the context in which adoption decisions would occur. In the case of the Dual Herbicide Sprayer, this involved consideration of pesticide runoff from sugarcane systems and its implications for GBR water quality. Residual herbicides such as Diuron persist in the environment and have been identified as a risk to reef ecosystems (Aaron Davis et al. 2016; Karim et al. 2023). Although industry initiatives and regulatory changes have reduced Diuron use over time, measurable concentrations continue to be detected in GBR waters (Skerratt et al. 2023).

Within this context, the Dual Herbicide Sprayer was promoted as a practice change capable of reducing residual herbicide use while maintaining agronomic performance (Robertson and Blair 2015). The TWG considered how these claimed benefits, together with the associated risks and management implications, would likely be perceived by non-adopting growers when responding to the ADOPT questions.

Grower workshops to introduce the dual-band sprayer were held in August–December of 2012, with demonstration trials in 2013 (Blair et al. 2019). By July 2016 the innovation was only used on about 3% of Queensland sugarcane (Blair et al. 2019).

### Adoption Characteristics of the Dual Herbicide Sprayer

The information presented below reflects what could reasonably be inferred about the Dual Herbicide Sprayer from publicly available sources. Such material forms part of the information environment within which farmers assess the practice. Adoption decisions are shaped by how benefits, risks, and costs are communicated and understood, making these sources directly relevant to the present analysis. The available literature (State of Queensland 2017; Blair et al. 2019), much of it produced by the technology developers, describes the core technical characteristics of the sprayer but provides limited contextual information about its performance under diverse farming conditions. While it identifies elements of potential relative advantage, it does not provide sufficient detail to support a full ADOPT analysis.

This literature suggests that environmental gains can be achieved without compromising agronomic performance. In particular, it indicates that reliance on residual PSII herbicides in sugarcane systems can be reduced without reductions in yield, quality, or crop safety through a technology that fits existing grower equipment, practices, and constraints. It also suggests that grower reluctance toward the technology has been driven more by perceived risk than by evidence (Blair et al. 2019; State of Queensland 2017).

Taken together, the literature relating to the Dual Herbicide Sprayer identifies several factors likely to influence adoption that align with key components of relative advantage as conceptualized in the ADOPT tool (Kuehne et al. 2017). Drawing on this material, the following ADOPT adoption influences can be inferred as particularly relevant to adoption outcomes for the Dual Herbicide Sprayer:

- Q 14: Relative upfront cost^2^
- Q 16: Profit benefit in years used
- Q 17: Future profit benefit
- Q 19: Environmental benefits
- Q 21: Risk
- Q 22: Ease & convenience

The *Relative up-front cost* (Q 14) of adopting the practice is around $3,000 (Thompson, 2013) which makes it a minor expense compared to other machinery costs on a sugarcane farm.

The *Economic benefits* (Q 16) gained from using the practice have been identified as decreased weed-control costs by using more of the low-cost herbicides such as glyphosate rather than newer, more expensive pre-emergent herbicides required to effectively control weeds (State of Queensland 2017). However, herbicides are only a small component of a sugarcane grower’s variable costs. They were 1.2% of variable costs in one scenario (Thompson 2013) means that changing to cheaper herbicides will have a minimal impact on variable costs. With a $3000 investment to modify an existing sprayer, the payback period is less than two years if 200 hectares are sprayed annually, which means that if only 28 hectares are treated each year, the break-even point stretches to 10 years (Thompson 2013).

*Future economic benefits* (Q17) can also occur. For example, when better weed control over time leads to fewer herbicide applications (State of Queensland 2017).

The *Environmental benefit* (Q 19) of using the Dual Herbicide Sprayer is that it allows glyphosate to be used for the spraying of the interrow, thereby reducing the use of residual herbicides by up to 55% (State of Queensland 2017; Thompson 2013).

A *Risk* (Q 21) that occurs from using a Dual Herbicide Sprayer is that it must be operated correctly. The windows of opportunity are narrower when applying contact and residual products simultaneously. Driver errors, such as momentary lapses in attention that cause deviation from the centerline of the crop row, can result in direct contact with sugarcane plants and significant crop damage. Also, there’s a higher risk of making mistakes when working with two chemicals than one. The additional chemical also adds complexity to the system. This is because it would only take a minor decline in yield (0.1%) to make the investment in the sprayer economically unviable (State of Queensland 2017; Thompson 2013).

*Ease and convenience* (Q 22) of using the Dual Herbicide Sprayer reduces as more actions need to be taken to manage risks. However, there are potentially some positive impacts on ease and convenience in that the decreased use of residuals means growers have a greater ability to comply with regulations (State of Queensland 2017; Thompson 2013). Growers may also achieve better weed control by treating the space between rows with glyphosate rather than conventional residual herbicide tank mixes (State of Queensland 2017) or being able to strategically target weeds if they occur (Thompson 2013). On the other hand, as already mentioned, the risk of damaging the sugarcane crop means greater attention is needed to spray application.

Taken together, the publicly available literature allows informed but limited inferences to be made about how the Dual Herbicide Sprayer aligns with key ADOPT adoption influences (Table 2). This material largely reflects the perspectives of practice proponents and as is common in the promotion of innovations, exhibits elements of optimism bias that can lead to unrealistic expectations regarding adoption outcomes (Rogers, 2003).

**Table 2:** Literature-based adoption influences for the Dual Herbicide Sprayer, interpreted using ADOPT

Q 14: Relative upfront cost	Minor relative upfront cost (compared to other machinery expenses).
Q 16: Profit benefit in years used	Negligible profit in the years that it is used (compared to the existing profit from growing sugarcane).
Q 17: Future profit benefit	Negligible future profit (from using it).
Q 19: Environmental benefits	Small or no environmental benefit (from the grower’s perceptions).
Q 21: Risk	Increased risk (that outweighs the benefits from its use).
Q 22: Ease & convenience	Reduced ease and convenience (because of the added complexity and the more careful management required).

However, because ADOPT requires structured, population-level judgements rather than generalized or promotional claims, this material alone is insufficient to generate disciplined adoption predictions. In particular, it does not clarify how a defined target population is likely to weigh these influences in practice or which constraints are most limiting.

**Fig. 4:**
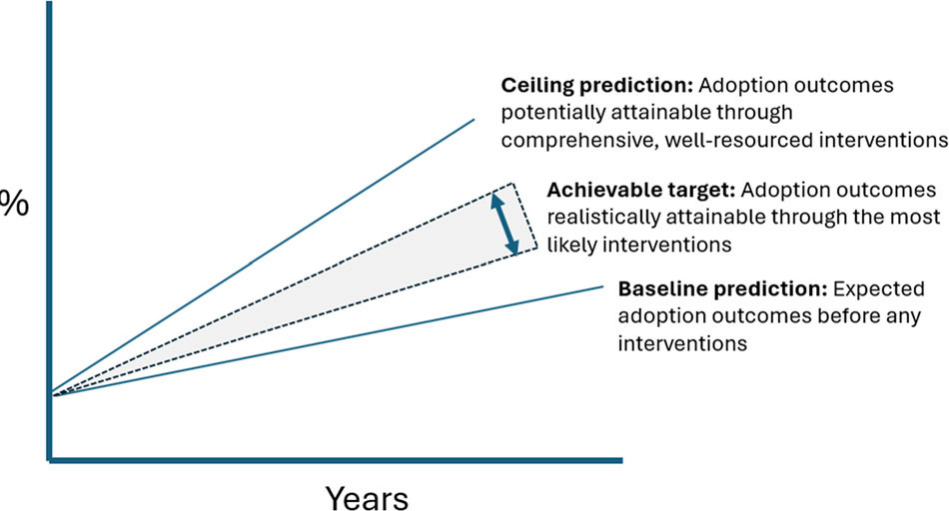
Baseline, achievable, and ceiling adoption predictions over time

To move beyond these constraints, we conducted a structured workshop with a Technical Working Group whose members were familiar with both the priority practice and the target population. This process involved generating a baseline prediction of adoption outcomes, identifying which adoption influences could plausibly be modified, and developing a ceiling prediction that reflected the potential effects of targeted, well-resourced interventions (Fig. 4).

### Baseline Analysis

The TWG suggested that, despite approximately 12 years of grower awareness, adoption of the Dual Herbicide Sprayer remained at around 2% of canegrowers. A baseline ADOPT analysis was therefore conducted, with the target population defined as the 98% of growers who had not adopted the technology.

**Fig. 5:**
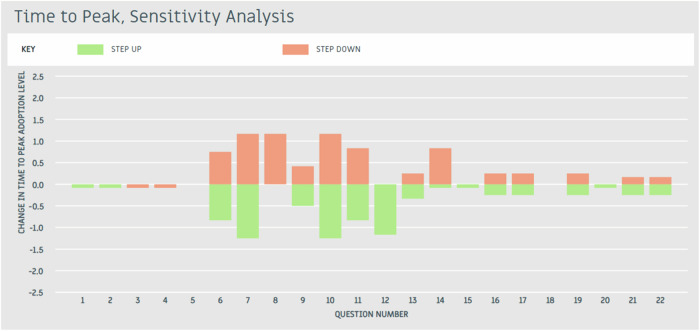
Time to peak adoption level sensitivity analysis

**Fig. 6:**
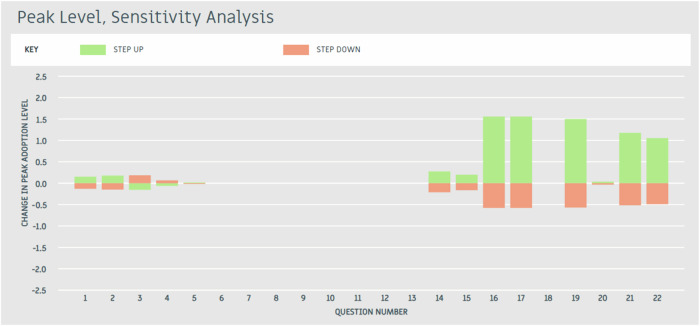
Peak adoption level sensitivity analysis

The baseline ADOPT results indicated a Peak Adoption Level of 2%, reached after 13 years. The drivers of these baseline adoption outcomes were then examined using ADOPT’s sensitivity analysis (Figs. 5 and 6). The adoption influences affecting the Time to Adoption were Questions 6: Short-term constraints, 7: Trialling ease, 8: Innovation complexity, 10: Advisory support, 11: Group involvement, and 14: Relative up-front cost (Fig. 5). The adoption influences affecting the Peak Adoption Level were Questions 16: Profit benefit in years used, 17: Future profit benefit, 19: Environmental benefits, 21: Risk, and 22: Ease and convenience (Fig. 6). These influential adoption factors were subsequently used to inform the design and testing of targeted interventions in the ceiling ADOPT analysis.

### Ceiling Analysis

The following subsection describes how the most influential adoption constraints identified in the baseline analysis were deliberately targeted through interventions and tested using a ceiling ADOPT analysis.

The TWG members were asked to identify which of the influences on adoption outcomes could realistically be changed and to specify the programmes and policies needed to effect changes. The changes that they identified as being practical were made to the baseline ADOPT responses. The revised adoption outcomes were framed as *ceiling* predictions, representing expected adoption outcomes if well-resourced interventions were comprehensively implemented. These estimates define an upper bound of *potentially* achievable adoption and are best understood as a conditional maximum rather than a forecast (Table 3).

**Table 3:** Changes to key drivers of time to peak adoption for dual herbicide sprayer

*Time to Peak Adoption (years)*	*Baseline prediction*	*Ceiling prediction*	*Potential intervention*	*Resource requirements*
	13	8		
*7. Trialable* How easily can the innovation (or significant components of it) be trialled on a limited basis before a decision is made to adopt it on a larger scale?	7.3 Moderately trialable	7.4 Easily trialable	Provide grower access to suitable equipment for trialling. Provide technical support to assist with trialling.	Loan machinery Technical experts
*8. Innovation complexity* Does the complexity of the innovation allow the effects of its use to be easily evaluated when it is used?	8.5 Not at all difficult to evaluate effects of use due to complexity	8.5 Not at all difficult to evaluate effects of use due to complexity	Unlikely to be changed.	
*10. Advisory support* What proportion of the target population uses paid advisors capable of providing advice relevant to the project?	10.3 About half use a relevant advisor	10.4 A majority use a relevant advisor	Provide capacity building for resellers and advisors. Make pesticide selection tool available and list of recommended pesticides.	Technical extension staff skilled in providing pesticide advice, or willing to be trained. Fully developed pesticide selection tool.
*12. Relevant existing skills & knowledge*. What proportion of the target population will need to develop substantial new skills and knowledge to use the innovation?	12.1 Almost all need new skills and knowledge	12.3 About half will need new skills and knowledge	Provide capacity building resellers and all advisors. Make pesticide selection tool available and list of recommended pesticides. Hold grower workshops to build grower capacity.	Extension staff skilled in providing pesticide advice. Workshop organizer and facilitator.
*14. Relative upfront cost of the project*. What is the size of the up-front cost of the investment relative to the potential annual benefit from using the innovation?	14.3 Moderate initial investment	14.4 Minor initial investment	Partial grant for spray technology and technical advice/audits. Economic analysis for fact sheets, case studies, and grower testimonials	Financial subsidy. Economic analyses. Publications assistance.

This can be better thought of as “advisors who act in the best interest of the client”

The TWG identified two distinct sets of adoption influences that could realistically be modified through policy or programme interventions. The first set primarily affected the speed of adoption, reducing the predicted Time to Peak Adoption from 13 years under the baseline scenario to 8 years under the ceiling scenario (Table 2). These influences related mainly to trialability, advisory support, existing skills and knowledge, and upfront cost, indicating that learning processes and early adoption barriers were the dominant constraints on adoption timing. For example, improving trialability through access to loan equipment and skilled operators reduced the time required for growers to assess the practice, while targeted advisory support addressed perceived implementation risks.

In contrast, a smaller set of influences affected the extent of adoption, increasing the predicted Peak Adoption Level from 2% to 18% under the ceiling scenario (Table 3). These influences were associated with future profitability, perceived environmental benefits, and risk exposure, highlighting the importance of how longer-term benefits and risks are understood and communicated by growers.

The following section reflects on the methodological insights arising from the baseline and ceiling analyses and considers their implications for policy and programme design.

## Discussion

### Methodological Insights and Policy Implications

The sequence of actions described represents a methodological refinement in the application of ADOPT, focused on how its inputs are generated—embedding structured, facilitated perspective-taking to improve the representativeness and robustness of the resulting predictions. While it is not possible to determine how ADOPT has been applied in all prior contexts, the explicit and systematically facilitated use of perspective-taking described here appears to represent a novel application of the tool.

Although ADOPT has been widely adopted as a tool for predicting farmer uptake of innovations (Kuehne et al. 2017), published applications typically provide little detail on how inputs are generated and rarely describe structured processes designed to ensure that responses are considered from the perspective of the target population. The present study contributes by formalizing and reinforcing perspective-taking as a methodological refinement within the ADOPT process.

This discussion reflects on the methodological insights arising from the series of ADOPT workshops and considers their implications for policy and programme design. One key insight concerns the effort required to sustain perspective-taking when applying population-level adoption prediction tools such as ADOPT.

Across the 15 regional workshops, each of which examined multiple priority practices within a given industry and catchment, TWG members demonstrated a growing awareness of the need for disciplined perspective-taking. This awareness strengthened as they progressed through the stages of applying the ADOPT process. Although participants initially attempted to respond from the perspective of the target population, this way of thinking was not yet habitual. Over the course of each workshop, ongoing reinforcement and facilitation were required to sustain it. By the later stages of discussion, participants were actively reminding one another to frame their responses from the standpoint of non-adopting growers.

This pattern suggests that perspective-taking is not automatic, particularly for participants who are themselves members of the industry under consideration, and that structured facilitation plays an important role in reducing self-referential thinking.

The ADOPT tool is designed to generate predictions of adoption outcomes for a target population rather than for individual farmers. Its application therefore requires users to assess how a defined population is likely to respond to a set of adoption influences, which involves deliberate perspective-taking beyond one’s own circumstances. Without structured support, there is a risk that responses may default to self-referential or normative assumptions, potentially weakening the reliability of the inputs. In this sense, facilitation is not merely procedural but helps ensure that the population-level predictions generated by ADOPT are grounded in disciplined and contextually informed judgment.

This experience reflects challenges identified in the literature, which show that maintaining another person’s perspective requires deliberate effort and adjustment from one’s own viewpoint (Mark Davis et al. 1996; Epley and Gilovich, 2001; Epley et al. 2004).

The initial difficulty TWG members experienced in maintaining a farmer-aligned perspective does not undermine the results; rather, it highlights the cognitive tendency toward self-referential judgment and underscores the importance of structured facilitation. By explicitly embedding perspective-taking within the process, the workshops were designed to mitigate this bias and strengthen the credibility of the resulting adoption assessments.

This process of identifying which influences on adoption outcomes can be modified clarifies the interventions, which could likely to be most effective and where potential synergies or overlaps with other adoption factors may enhance their impact. The caveat is that the ceiling adoption outcomes are those which could be expected if sufficient resources were expended to convincingly achieve all of the changes identified in Tables 3, 4. With this amount of effort, the predicted improvements for this case example are significant: the Peak Adoption Level could rise from 2 to 18%, and the Time to Peak Adoption could reduce from 13 years down to 8 years. However, achieving these changes is unlikely to occur without major interventions, that could include financial subsidies, targeted incentives, or strategies aimed at shifting farmers’ perceptions of the benefits gained from the Dual Herbicide Sprayer. These types of changes are difficult to bring about and cannot be assumed as straightforward. They may be expensive, time-consuming, and limited by the availability of human and financial resources. In practice, the achievable target adoption rate is likely to fall somewhere between the baseline and the ceiling predictions.

**Table 4:** Changes to key drivers of peak adoption level for dual herbicide sprayer

*Peak Adoption Level (%)*	*Baseline prediction*	*Ceiling prediction*	*Potential intervention*	*Resource requirements*
	2%	18%		
*16. Profit benefit in years that it is used*. To what extent is the use of the innovation likely to affect the profitability of the farm business in the years that it is used?	16.5 Small profit advantage in years that it is used	16.5 Small profit advantage in years that it is used	Unlikely to be changed.	
*17. Future profit benefit*. To what extent is the use of the innovation likely to have additional effects on the future profitability of the farm business?	17.4 No profit advantage or disadvantage in the future	17.5 Small profit advantage in the future	Provide benefits of Dual Herbicide Sprayer through: Fact sheets, etc. on benefits from combatting resistance and effect on seed bank. Ongoing information on chemistry.	Pesticide specialist. Publications assistance.
*19. Environmental costs & benefits*. To what extent would the use of the innovation have net environmental benefits or costs?	19.5 Small environmental advantage	19.6 Moderate environmental advantage	Education programmes on benefits from avoiding resistance and effect on seed ban**k**.	Pesticide specialist. Publications assistance.
*21. Risk exposure*. To what extent would the use of the innovation affect the net exposure of the farm business to risk?	21.2 Moderate increase in risk	21.3 Small increase in risk	Education programmes on benefits from avoiding resistance and effect on seed bank. Demonstration trials to show best practice implementation.	Pesticide specialist. Publications assistance. Extension support.
*22. Ease and convenience*. To what extent would the use of the innovation affect the ease and convenience of the management of the farm in the years that it is used?	22.3 Small decrease in ease and convenience	22.3 Small decrease in ease and convenience	Unlikely to be changed.	

Policies informed by adoption predictions that incorporate a perspective-taking approach are more likely to generate interventions that are better aligned with farmers’ decision-making processes. By focusing on modifiable drivers of adoption, policymakers can design targeted strategies that reduce barriers to adoption. Directing resources toward interventions with more favourable adoption outcomes supports the more efficient use of public funds while maintaining grower productivity and achieving environmental outcomes.

Using ADOPT in a situated manner meant that workshop participants’ responses to the questions were more closely aligned with how the target group of farmers might have responded. While absolute accuracy with tools such as ADOPT is unattainable this approach provides a more rigorous and well-informed estimate of future adoption outcomes. The key was encouraging the TWG to develop and maintain an awareness of farmers’ perspectives, ensuring that their responses to the ADOPT questions might have reflected local contexts and real-world circumstances.

This study demonstrates how a structured, participatory application of an adoption prediction tool can be used to complement biophysical prioritisation frameworks by explicitly integrating adoption considerations into the design of water quality interventions.

### Implications for GBR Policy and Programme Design

The findings of this study have several implications for the design and implementation of policies and programmes aimed at improving water quality in the Great Barrier Reef catchment.

Incorporating perspective-taking into the prioritisation of practices can strengthen the legitimacy of GBR programmes by grounding biophysical objectives in the practical decision-making constraints faced by farmers. This helps demonstrate that priority practices are not selected solely on technical grounds, but also with regard to their feasibility and likely uptake—an important consideration for policy credibility in complex agricultural contexts.

The use of baseline and ceiling adoption outcome scenarios provides a transparent basis for setting realistic adoption targets. Rather than relying on overly optimistic assumptions of full or rapid uptake of priority practices, this approach aligns expectations with plausible adoption trajectories, reducing the risk of setting targets that are unachievable within programme timeframes.

By identifying the adoption influences that most strongly constrain uptake, the approach supports more efficient allocation of resources by focusing investment on interventions most likely to improve adoption outcomes. From a policy perspective, it also provides a transparent mechanism for linking practice prioritisation with intervention design. Rather than assuming that priority practices will be adopted once identified, the method allows policymakers to explore how different combinations of extension, incentives, or regulatory settings are likely to influence adoption trajectories. This is particularly relevant for GBR water quality initiatives, where investment effectiveness depends not only on selecting the “right” practices, but on ensuring that those practices are adopted broadly enough, and within appropriate timeframes, to meaningfully contribute to water quality objectives.

Farmers and industry participants are more likely to engage constructively with GBR programmes when priorities and interventions are informed by on-farm realities, rather than shaped primarily by top-down targets or best-practice prescriptions that do not fully reflect local farming contexts.

### Broader Applicability of the Refined ADOPT Approach

The refined ADOPT approach has broader applicability beyond the GBR case study because many agricultural environmental programmes globally face a similar public–private benefit gap. In these situations, practices often deliver public environmental benefits but provide only limited or uncertain private returns to farmers, leading to adoption targets that are optimistic and behaviourally unrealistic. The approach described here is applicable across diverse farming systems, including broadacre, horticultural, grazing, climate mitigation, and smallholder contexts, and is particularly useful where empirical adoption data are limited or costly to obtain. Importantly, the refinement not only predicts likely adoption outcomes but also helps identify which interventions are most influential in shaping uptake and therefore where policy effort is best directed. Its effective application, however, depends on knowledgeable participants, structured facilitation, and careful specification of the innovation and target population.

To our knowledge, this constitutes a novel application of a structured, theory-based adoption prediction tool within environmental programme design. Although specific drivers and intervention mixes will vary across environmental management challenges, industries, and national contexts, the underlying principles of ADOPT remain applicable, including in settings with more limited institutional or financial capacity. In addition, the peer-to-peer learning generated through structured ADOPT processes may contribute to behavioural change, particularly in contexts with limited extension capacity, by improving shared understanding of the longer-term economic and risk implications of practice change.

## Conclusion

Improving water quality in the Great Barrier Reef catchment remains constrained by uneven and often limited adoption of best management practices by farmers. Addressing this challenge requires approaches that integrate scientific assessments of water quality benefits with a realistic understanding of how farmers perceive and respond to proposed practice changes.

Where the consequences are substantial, such as the health of the GBR, a sound understanding of the likely adoption outcomes of priority practices is essential. To guide the identification and development of interventions to improve adoption outcomes, the ADOPT tool needs to be applied with methodological rigour so that its results are both empirically defensible and practically robust. This, in turn, requires that the inputs to the tool as interpreted by industry experts through perspective-taking reflect the realities faced by farmers when considering adoption of the priority practice.

The application of ADOPT described in this article represents our attempt to ensure a more systematic and robust approach. By following a guided process that emphasized identifying the key drivers of adoption and employing perspective-taking, the most effective interventions to enhance adoption could be identified. Developing realistic practice change targets when resources are limited will always be challenging. This application of ADOPT demonstrates that a more rigorous, farmer-aligned process is achievable, offering a stronger foundation for effective interventions and enduring practice change. While developed in the context of GBR water quality programmes, the approach is likely to be transferable to other policy settings where adoption targets are being set under conditions of limited data and high consequence.

## Supplementary information

Dual Herbicide Sprayer Ceiling Adoption Report

## Data Availability

No datasets were generated or analysed during the current study.

## Footnotes

1 ADOPT is a web-based tool accessible from: https://adopt.csiro.au

2 These are the question numbers and names used in the ADOPT tool.
