# Supplementary material for: A Methodological Refinement for Evaluating Adoption of Priority Practices for Water Quality Improvement in Australia’s Great Barrier Reef
Source: Environ Manage. 2026 Apr 21;76(5):167. doi: 10.1007/s00267-026-02449-6 (PMC13099839; doi:10.1007/s00267-026-02449-6)
Supplement: Supplementary file 1 — Dual Herbicide Sprayer Ceiling Adoption Report [file 267_2026_2449_MOESM1_ESM.pdf]

The adoption and diffusion outcome prediction tool

Adoption report for:
DUAL HERBICIDE SPRAYER (INGHAM)

Report Authors:
Ingham Sugar Cane Technical Working Group

10/01/2026

For more information about ADOPT contact adopt@csiro.au

Project Details

MODEL
Standard Agriculture

YOUR INNOVATION
Dual Herbicide Sprayer

YOUR POPULATION
Cane Growers

Adoption Level

TIME TO NEAR-PEAK ADOPTION LEVEL
(years)

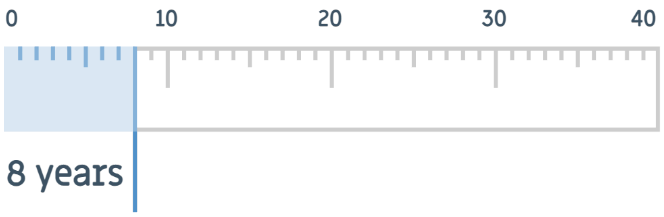

PEAK ADOPTION LEVEL
(percent %)

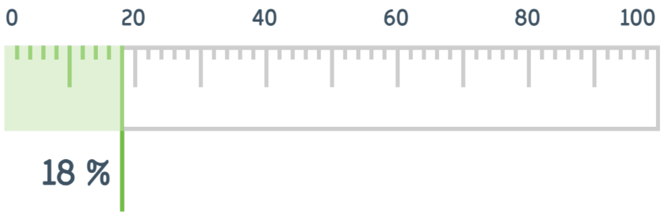

Predicted Adoption Levels

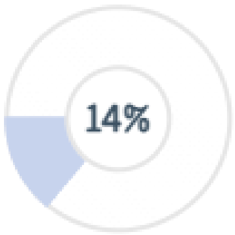

IN 5 YEARS FROM START

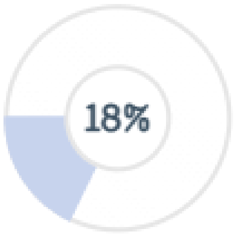

IN 10 YEARS FROM START

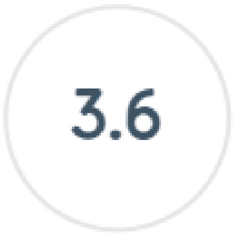

TIME TO 50% OF PEAK ADOPTION (years)

NOTES: The predictions of Peak Adoption Level and Time to Peak Adoption Level are numeric outputs that are provided to assist with insight and understanding and like any forecasts should be used with caution. Time to Near Peak Adoption represents the time to 99% of the maximum predicted adoption level.

Adoption Level S-Curve

The following chart shows how the level of adoption in the relevant population of farmers changes over time.

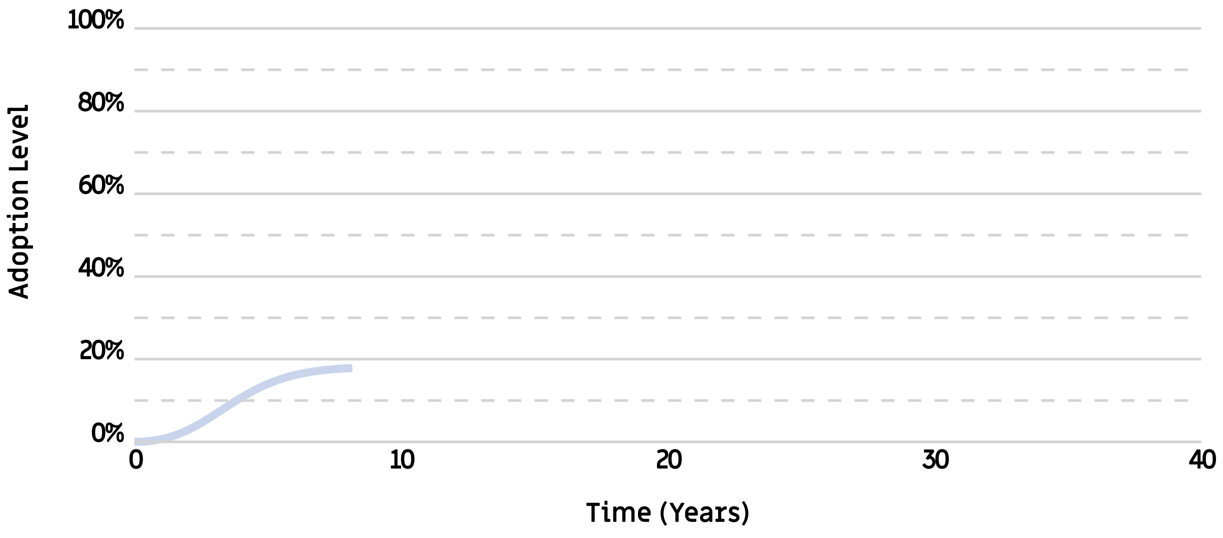

Yearly Adoption Levels

Year	Adoption %
1	1
2	3
3	7
4	11
5	14
6	16
7	17
8	18

(Peak Adoption)

Changing the adoption levels

Many of the factors can be changed by activities such as extension. Based on the data entered, the ADOPT model suggests that changing the following factors would have the biggest effect on adoption.

Changing the peak adoption level

MOST SENSITIVE QUESTION

16 Profit benefit in years that it is used

To what extent is the use of the innovation likely to affect the profitability of the farm business in the years that it is used?

YOUR RESPONSE

Small profit advantage in years that it is used

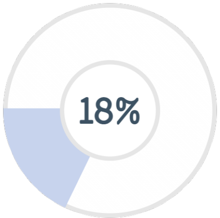

STEP UP RESPONSE

Moderate profit advantage in years that it is used

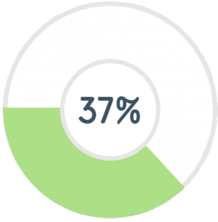

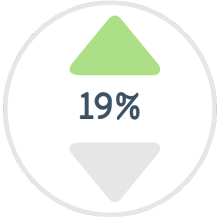

STEP DOWN RESPONSE

No profit advantage or disadvantage in years that it is used

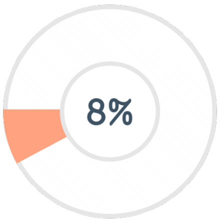

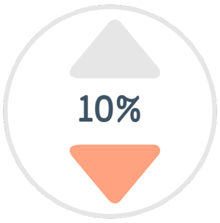

Changing the time to peak adoption level

MOST SENSITIVE QUESTION

7 Trialable

How easily can the innovation (or significant components of it) be trialled on a limited basis before a decision is made to adopt it on a larger scale?

YOUR RESPONSE

Easily trialable

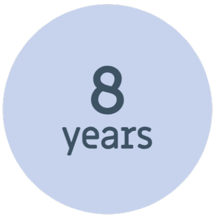

STEP UP RESPONSE

Very easily trialable

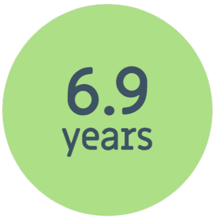

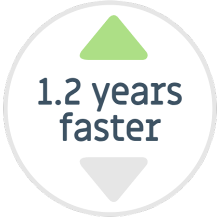

STEP DOWN RESPONSE

Moderately trialable

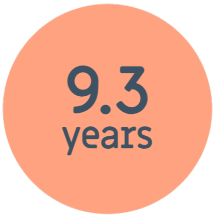

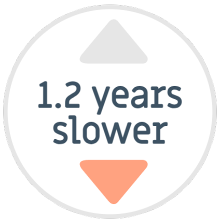

Sensitivity Analysis

The following charts show the effects on Peak Adoption Level and Time to Peak Adoption of single step changes up and down for all questions.

Peak level, sensitivity analysis

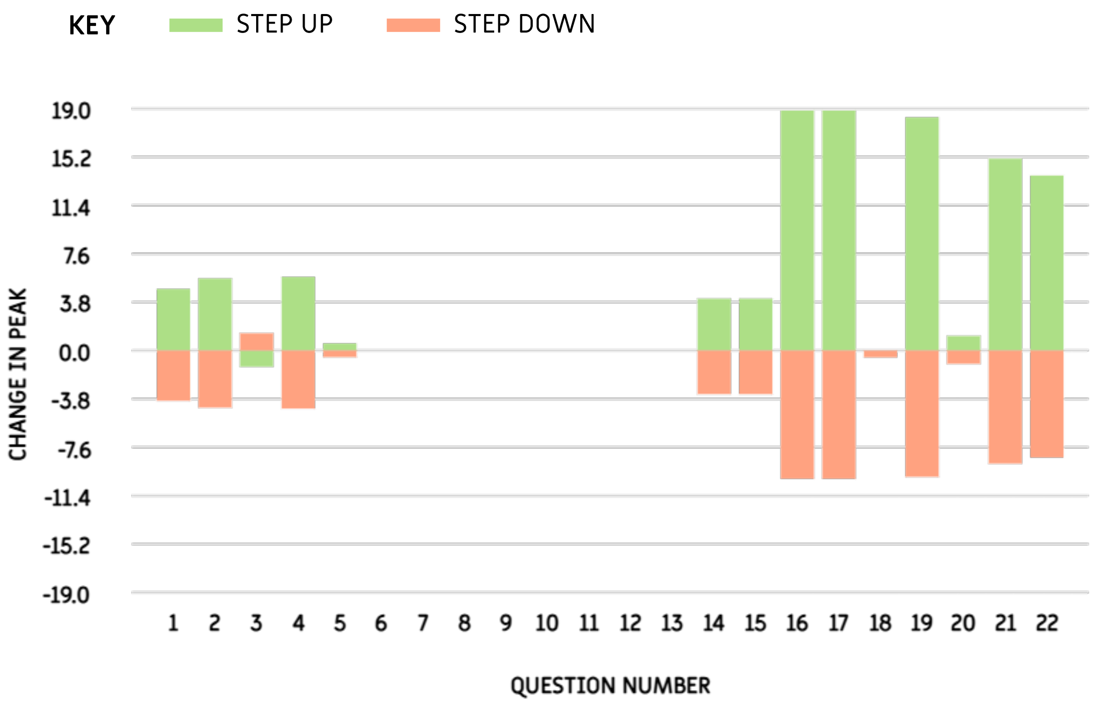

Time to peak, sensitivity analysis

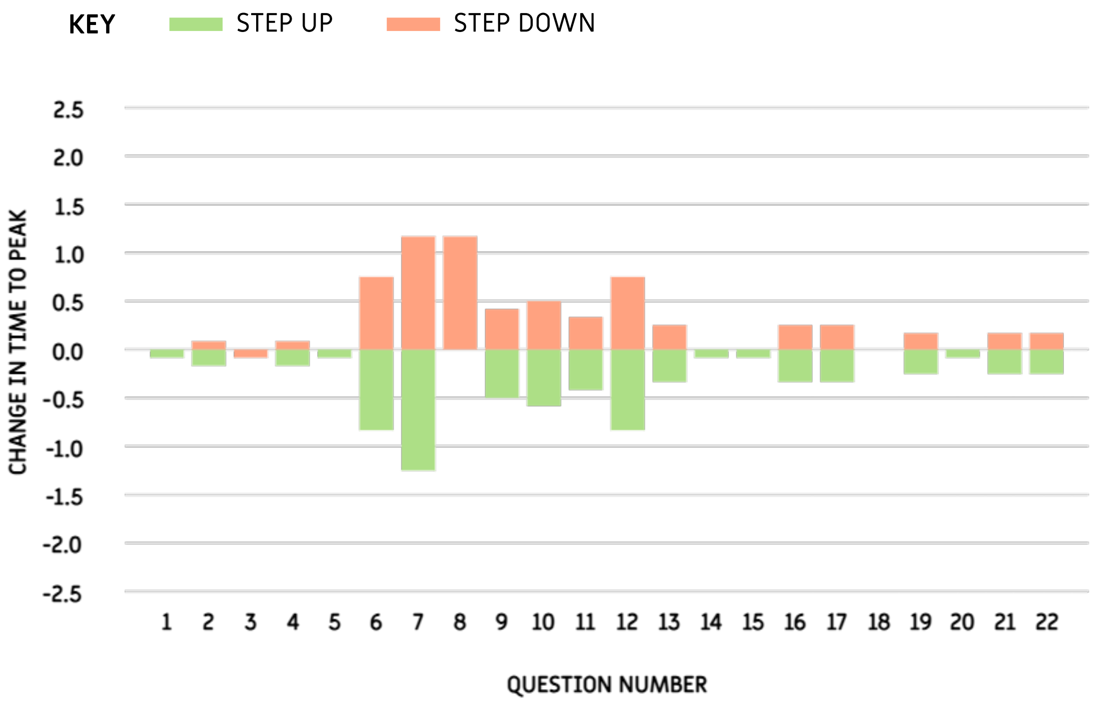

S-Curve Sensitivity

The following chart shows how the S-Curve is predicted to change when a single step change is made to the most sensitive question(s) with respect to Peak Adoption Level

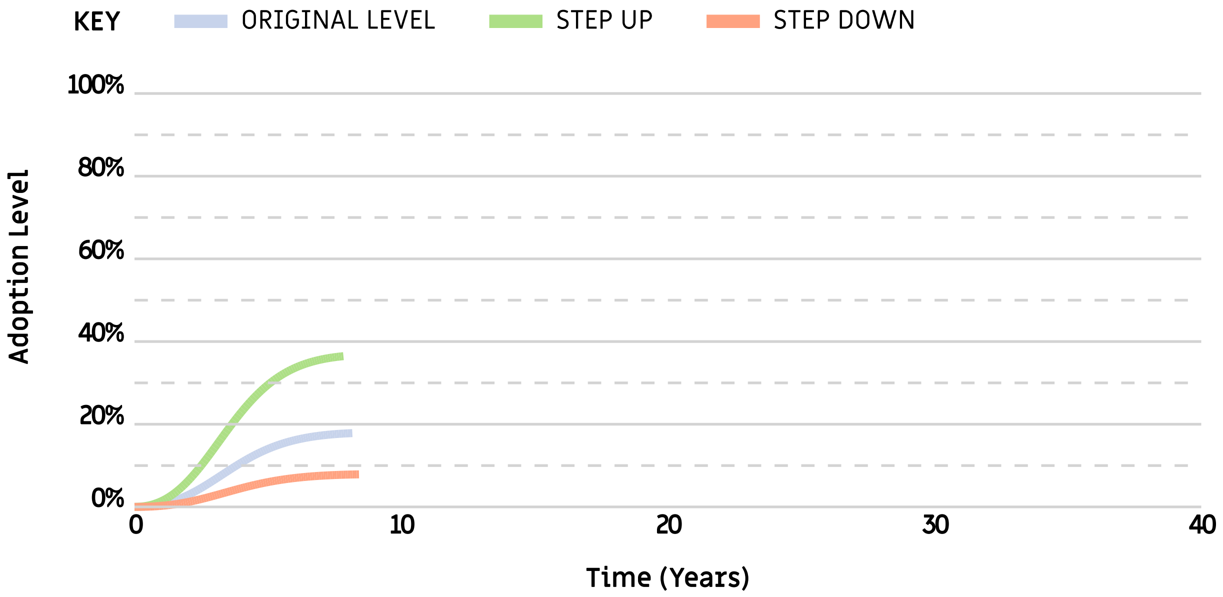

The following chart shows how the S-Curve is predicted to change when a single step change is made to the most sensitive question(s) with respect to Time to Near Peak Adoption

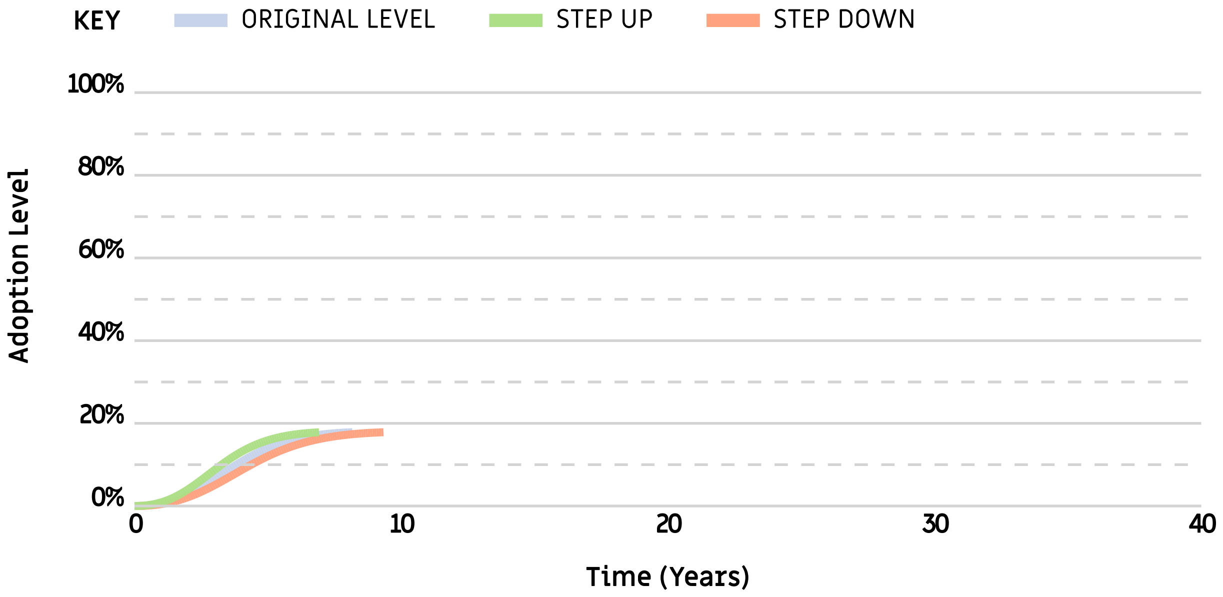

Sensitivity Analysis Table

Question	Peak Level (%)		Time to Peak (years)	
	Impact of 1 step up	Impact of 1 step down	Impact of 1 step up	Impact of 1 step down
1	4.83	-3.95	-0.08	0
2	5.65	-4.48	-0.17	0.08
3	-1.27	1.35	0	-0.08
4	5.78	-4.56	-0.17	0.08
5	0.55	-0.52	-0.08	0
6	0	0	-0.83	0.75
7	0	0	-1.25	1.17
8	n/a	0	n/a	1.17
9	0	0	-0.5	0.42
10	0	0	-0.58	0.5
11	0	0	-0.42	0.33
12	0	0	-0.83	0.75
13	0	0	-0.33	0.25
14	4.07	-3.42	-0.08	0
15	4.07	-3.42	-0.08	0
16	18.85	-10.07	-0.33	0.25
17	18.85	-10.07	-0.33	0.25
18	n/a	-0.54	n/a	0
19	18.31	-9.92	-0.25	0.17
20	1.14	-1.04	-0.08	0
21	15.06	-8.88	-0.25	0.17
22	13.74	-8.4	-0.25	0.17

Responses

Question	Response	Reasoning
**Relative Advantage for the Population**		
1. Profit orientation	A majority have maximising profit as a strong motivation	
2. Environmental orientation	About half have protection of the environment as a strong motivation	
3. Risk orientation	A majority have risk minimisation as a strong motivation	
4. Enterprise scale	A majority of the target farms have a major enterprise that could benefit	
5. Management horizon	A majority have a long-term management horizon	
6. Short term constraints	About half currently have a severe short-term financial constraint	
**Learnability Characteristics of the Innovation**		
7. Trialable	Easily trialable	Potential intervention: Provide grower access to suitable equipment for trialling. Provide technical support to assist with trialling. Resource requirements: Loan machinery, Technical experts
8. Innovation complexity	Not at all difficult to evaluate effects of use due to complexity	
9. Observability	Easily observable	
**Learnability of Population**		

10. Advisory support	A majority use a relevant advisor	Potential intervention: Provide capacity building for resellers and advisors. Make pesticide selection tool available and list of recommended pesticides. Resource requirements: Technical extension staff skilled in providing pesticide advice, or willing to be trained. Fully developed pesticide selection tool.
11. Group involvement	About half are involved with a group that discusses farming	
12. Relevant existing skills & knowledge	About half will need new skills and knowledge	Potential intervention: Provide capacity building resellers and all advisors. Make pesticide selection tool available and list of recommended pesticides. Hold grower workshops to build grower capacity. Resource requirements: Extension staff skilled in providing pesticide advice. Workshop organiser and facilitator.
13. Innovation awareness	About half are aware that it has been used or trialed in their district	
**Relative Advantage of the Innovation**		
14. Relative upfront cost of the project	Minor initial investment	Potential intervention: Partial grant for spray technology and technical advice/audits. Economic analysis for fact sheets, case studies, and grower testimonials. Resource requirements: Financial subsidy. Economic analyses. Publications assistance.
15. Reversibility of the innovation	Moderately difficult to reverse	

16. Profit benefit in years that it is used	Small profit advantage in years that it is used	
17. Future profit benefit	Small profit advantage in the future	Potential intervention: Provide benefits of Dual Herbicide Sprayer through: Fact sheets, etc on benefits from combatting resistance and effect on seed bank. Ongoing information on chemistry. Resource requirements: Pesticide specialist. Publications assistance.
18. Time until any future profit benefits are likely to be realised	Not Applicable	
19. Environmental costs & benefits	Moderate environmental advantage	Potential intervention: Education programs on benefits from avoiding resistance and effect on seed bank. Resource requirements: Pesticide specialist. Publications assistance.
20. Time to environmental benefit	Immediately	
21. Risk exposure	Small increase in risk	Potential intervention: Education programs on benefits from avoiding resistance and effect on seed bank. Demonstration trials to show best practice implementation. Resource requirements: Pesticide specialist. Publications assistance. Extension support.
22. Ease and convenience	Small decrease in ease and convenience	

ADOPT can be cited as: Kuehne G, Llewellyn R, Pannell D, Wilkinson R, Dolling P, Ouzman J, Ewing M (2017) Predicting farmer uptake of new agricultural practices: A tool for research, extension and policy, Agricultural Systems 156:115-125- https://doi.org/10.1016/j.agsy.2017.06.007

While CSIRO makes every effort to ensure that the information on this site (including the ADOPT tool and associated- materials) is accurate, current and complete, CSIRO makes no representations, conditions or warranties of any kind, express or implied, as to the operation or results of this site, or accuracy, correctness or reliability of the information available on this site. The information provided is subject to the usual uncertainties of research and does not constitute expert advice. Users should not rely solely on any of the information provided. To the maximum extent permitted by law, CSIRO does not guarantee the completeness or accuracy of any of the information contained on or accessed through this site and excludes all liability to any person arising directly or indirectly from using this site and any information or material available on it.

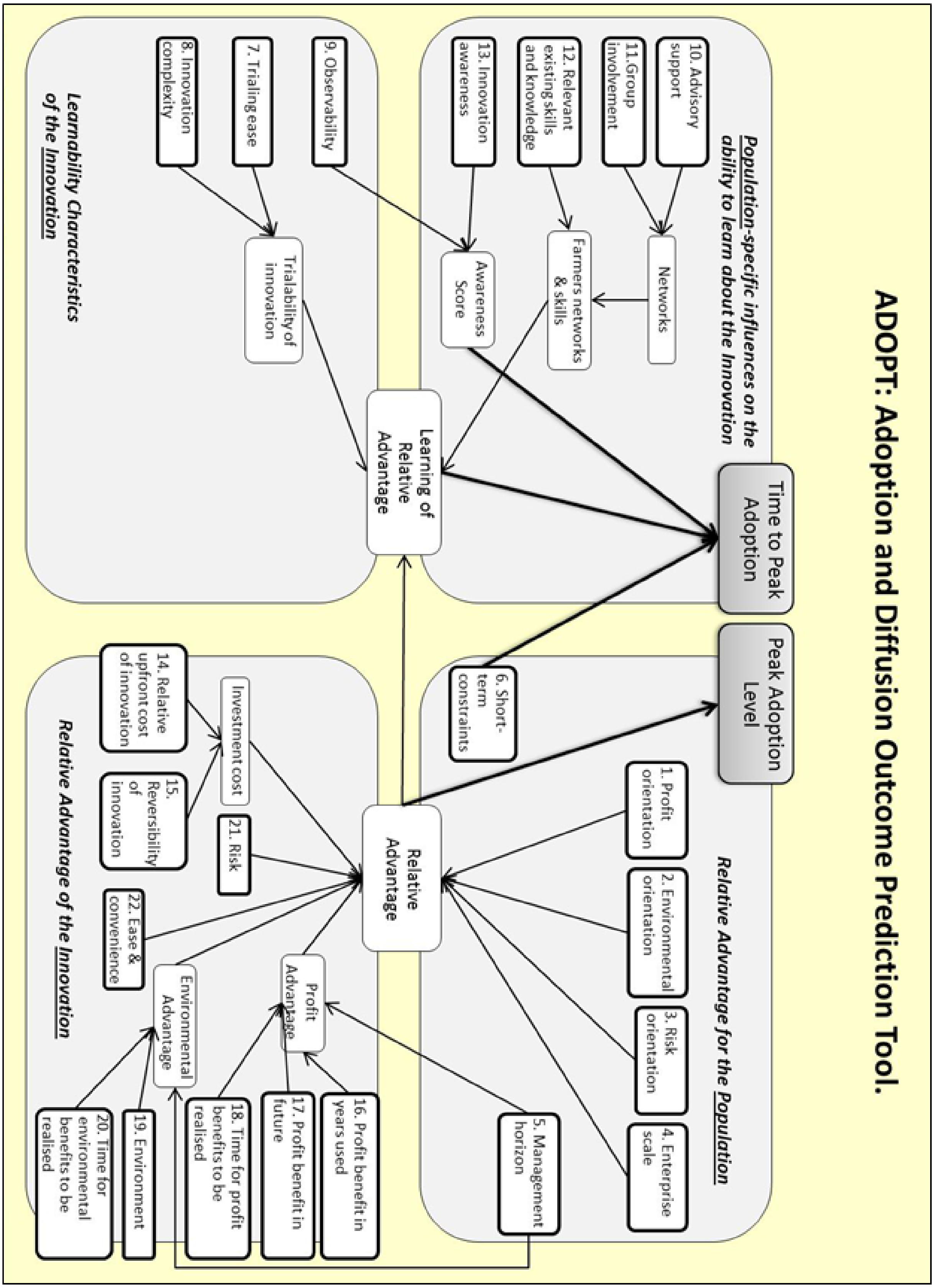

Copyright © CSIRO 2026
